# Sodium Ion‐Induced Structural Transition on the Surface of a DNA‐Interacting Protein

**DOI:** 10.1002/advs.202401838

**Published:** 2024-09-20

**Authors:** Chunhua Xu, Yue Lu, Yichao Wu, Shuaikang Yuan, Jianbing Ma, Hang Fu, Hao Wang, Libang Wang, Hao Zhang, Xuan Yu, Wei Tao, Chang Liu, Shuxin Hu, Yi Peng, Wenfei Li, Yunliang Li, Ying Lu, Ming Li

**Affiliations:** ^1^ Beijing National Laboratory for Condensed Matter Physics, Institute of Physics Chinese Academy of Sciences Beijing 100190 China; ^2^ Songshan Lake Materials Laboratory Dongguan Guangdong 523808 China; ^3^ Wenzhou Key Laboratory of Biophysics, Wenzhou Institute University of Chinese Academy of Sciences Wenzhou Zhejiang 325000 China; ^4^ School of Physics Nanjing University Nanjing 210093 China; ^5^ University of Chinese Academy of Sciences Beijing 100049 China

**Keywords:** protein surface, salt‐induced structural transition, single‐molecule thermodynamics, single‐stranded DNA binding protein, structural dynamics

## Abstract

Protein surfaces have pivotal roles in interactions between proteins and other biological molecules. However, the structural dynamics of protein surfaces have rarely been explored and are poorly understood. Here, the surface of a single‐stranded DNA (ssDNA) binding protein (SSB) with four DNA binding domains that bind ssDNA in binding site sizes of 35, 56, and 65 nucleotides per tetramer is investigated. Using oligonucleotides as probes to sense the charged surface, NaCl induces a two‐state structural transition on the SSB surface even at moderate concentrations. Chelation of sodium ions with charged amino acids alters the network of hydrogen bonds and/or salt bridges on the surface. Such changes are associated with changes in the electrostatic potential landscape and interaction mode. These findings advance the understanding of the molecular mechanism underlying the enigmatic salt‐induced transitions between different DNA binding site sizes of SSBs. This work demonstrates that monovalent salt is a key regulator of biomolecular interactions that not only play roles in non‐specific electrostatic screening effects as usually assumed but also may configure the surface of proteins to contribute to the effective regulation of biomolecular recognition and other downstream events.

## Graphical abstract

Cationic and anionic residues on protein surfaces are often inter‐connected into hydrogen bond networks to shape protein–ligand interactions. Here it is found that sodium ions not only exhibit electrostatic screen effects but also induce discontinuous structural transition on the protein surface. The transition is instrumental in altering the wrapping of single‐stranded DNA on SSB, a key protein in DNA replication, recombination, and repair.

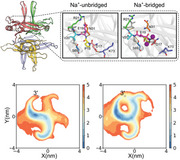

## 1 Introduction

Protein surfaces play pivotal roles in almost all biological processes involving protein interactions. The binding of DNA to proteins provides a paradigm for studying protein surfaces.^[^ ^1^ ^]^ Many cationic groups on protein surfaces that form ion pairs with DNA phosphates can form hydrogen bonds and/or dehydrated salt bridges (hydrogen‐bonded ion pairs) with neighboring anionic side chains.^[^ ^1^, ^2^ ^]^ Patterns defined by the hydrogen bonds and/or salt bridges may therefore dictate protein–DNA interactions.^[^ ^3^ ^]^ Single‐stranded DNA (ssDNA) binding proteins (SSBs) protect ssDNA from nucleases and prevent the formation of secondary structures in ssDNA. *Escherichia coli* SSB forms stable homo‐tetramers at sub‐nanomolar concentrations and its four DNA binding domains bind ssDNA in binding site sizes of 35, 56, and 65 nucleotides (nt) per tetramer, and are referred to as (SSB)_35_, (SSB)_56,_ and (SSB)_65_ binding modes, respectively.^[^ ^4^ ^]^ The transitions and related thermodynamics of these binding modes have been examined extensively.^[^ ^4^, ^5^ ^]^ The binding of SSB to ssDNA is influenced by both the concentration and type of various cations and anions. Under conditions of high ionic strength (>200 mm NaCl) and low SSB–ssDNA ratios, the (SSB)_65_ mode is favored, whereas under low ionic strengths and high SSB–ssDNA ratios, the (SSB)_35_ mode is favored.^[^ ^4^, ^5^ ^]^ Divalent cations, such as Mg^2+^ and Ba^2+^, are more effective in promoting transitions between the binding modes than monovalent ions.^[^ ^4^, ^5^, ^6^ ^]^ In alkaline environments (pH 8.1), the type of anion (Cl^−^ vs Br^−^) does not impact the binding mode transitions, whereas in acidic environments (pH 6.5), anions significantly influence mode transitions.^[^ ^4^, ^5^, ^6^ ^]^ Moreover, negative cooperativity occurs between the ssDNA binding sites and is associated with binding mode transitions.^[^ ^4^, ^6^, ^7^ ^]^ Single molecular methods are powerful tools that have helped in the unveiling of the wrapping/unwrapping pathways of ssDNA from SSB.^[^ ^8^ ^]^ Complete unbinding of SSB at high forces was observed using optical tweezers.^[^ ^8c^ ^]^ Computationally, it was shown that both electrostatic and aromatic interactions are essential for effective binding between ssDNA and SSB.^[^ ^8d,e,g^ ^]^ Despite the intensive studies,^[^ ^5^, ^8^, ^9^ ^]^ it is still not clear how charged residues on the protein surface are affected by salts to dictate binding mode transitions.

High salt concentrations induce global structural transitions of proteins,^[^ ^10^ ^]^ mainly through the interactions of ions on the protein surface. Historically, studies on the effects of salts have centered predominantly on secondary or tertiary structures at the domain level. This approach overlooks the significant influence of salts on the charged residues on or near the protein surface that are usually direct participants in different molecular events. However, there is no direct evidence about how low concentrations of monovalent salts such as NaCl might induce structural transitions on the surfaces of water‐soluble proteins.

Structural dynamics of protein surfaces have rarely been explored and remain largely unknown. In this study, we chose SSB as a prototypical DNA‐interacting protein to study the structural dynamics of protein surfaces. We found that sodium ions induced cooperative reorganization of the network of hydrogen bonds and/or salt bridges on the protein surface. The findings provide clear evidence that a protein surface undergoes two‐state structural transitions in salt solutions, which deepens our understanding of the molecular mechanism underlying the enigmatic salt‐induced transitions between the different DNA binding site sizes of SSB.

## 2 Results and Discussion

### 2.1 Magnetic Tweezers Analysis of SSB Binding to 20‐nt ssDNA

**Figure 1:**
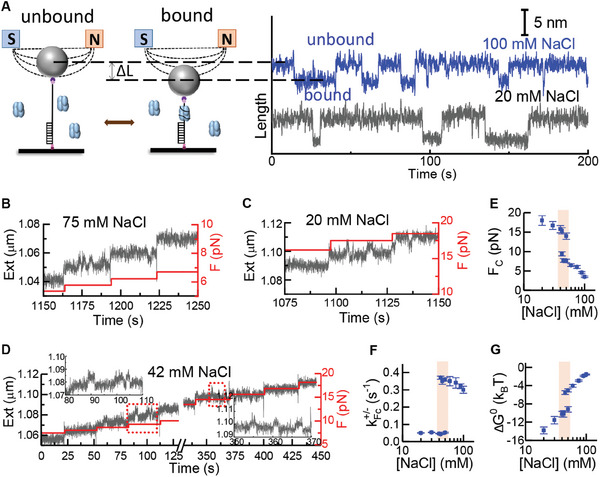
Magnetic tweezers assay of the binding/unbinding kinetics and strength of the DNA–protein interactions. A) Schematic representation of the experiments. Jumps of the extension of DNA are observed when the force is adjusted to the values at which the binding/unbinding of single‐stranded DNA binding protein (SSB) occurs. B,C) Typical time traces of the DNA extension (black lines) at different forces (red lines) at 75 mm and 20 mm NaCl. The repeated jumps at certain forces in the traces indicate the binding/unbinding of single SSB molecules. D) At 42 mm NaCl, two types of repeated jumps occur, one at ≈10 pN and another at ≈15 pN. Insets show details of the black lines in the red dotted rectangles. SSB concentration = 10 nM. E–G) Thermodynamic parameters versus NaCl concentration for SSB binding to the single‐stranded DNA: (E) Critical forces; (F) Binding or unbinding rates at the critical forces; (G) Binding free energies at zero force. Error bars are standard errors.

The shortest ssDNA segment that is relevant to the binding mode transitions is ≈17‐nt long.^[^ ^8c^ ^]^ We used magnetic tweezers to measure the binding and unbinding of SSB to a 20‐nt ssDNA (**Figure** **1**A, left). We used the slightly longer 20‐nt ssDNA to avoid potential interferences with the 3′ and 5′ double‐stranded DNA handles, and on the other hand, the 20‐nt ssDNA is much shorter than the SSB binding site size (35 nt) in the low salt concentration range,^[^ ^4^ ^]^ it allowed us to simplify the data analysis. We observed jumps of positions of the magnetic bead when the force was adjusted to values at which unbinding of SSB occurs. The binding of a new SSB again reduced the extension of the ssDNA.^[^ ^11^ ^]^ The long DNA extensions indicate the unbound state and the short DNA extensions indicate the bound state (Figure 1A, right). The range of the force required to observe the jumps depended on the NaCl concentration. For example, for 75 mm NaCl, jumps were observed at ≈7 pN (Figure 1B), whereas for 20 mm NaCl, jumps were observed at ≈15 pN (Figure 1C). Interestingly, at the intermediate 42 mm NaCl concentration, jumps were observed near two forces: one at ≈10 pN and the other at ≈15 pN (Figure 1D). A similar binding/unbinding pattern with larger jumps of the DNA extension was observed when a 70 nt ssDNA was used in the experiments (Figure S1A, Supporting Information). A control experiment was performed without SSB using the DNA construct (ssDNA with two dsDNA handles). No discontinuous structural transition was observed at the salt concentrations that we studied (Figure S1B, Supporting Information).

The kinetics of binding and unbinding were derived from the time traces of the DNA extension (Figure S2A, Supporting Information). The inverse values of the characteristic dwell times (*t*_on_ and *t*_off_) (Figure S2B,C, Supporting Information) were the (observed) binding and unbinding rates, respectively. To exclude the possibility that the reduction in distance was because of the rewrapping of the ssDNA on the same SSB tetramer, we varied the SSB concentration and found that the binding rate increased linearly with SSB concentration, whereas the unbinding rate was independent of the SSB concentration (Figure S3A, Supporting Information). Similar binding/unbinding events were observed in an optical tweezer assay.^[^ ^8c^ ^]^ In our experiments, the force was carefully tuned to a critical value, *F*_c_, at which the binding/unbinding became balanced. We found that the binding rate decreased and the unbinding rate increased with force as shown in Figure S3B (Supporting Information). Therefore, *F*_c_ can be readily determined from the crossover of the two force‐dependent curves.

Overall, *F*_c_ decreased when the NaCl concentration increased because of the electrostatic screening effect (Figure 1E).^[^ ^6^, ^9^ ^]^ However, *F*_c_ jumped down near the transition midpoint. Using Equations (1) and (2) in the Method section, we calculated the null‐force binding free energy as a function of the NaCl concentration and found that it jumped up by ≈4.1 *k_B_T* near the transition midpoint (Figure 1G). The results suggest that two different interaction modes exist between the SSB protein and the 20‐nt ssDNA in NaCl solutions of moderate concentrations.

### 2.2 Infrared Analyses of Free SSB in Various NaCl Concentrations

**Figure 2:**
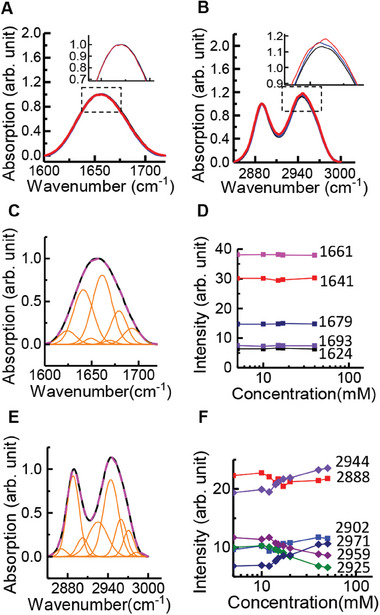
Fourier transform infrared (FTIR) spectra of SSB in NaCl/D_2_O solutions. A) Spectra in the amide I′ window 1600–1720 cm^−1^ at 5 mm (black line), 17 mm (blue line), and 40 mm (red line) NaCl concentrations. B) Spectra in the CH stretch window 2880–3000 cm^−1^. Full FTIR spectra at various NaCl concentrations are displayed in Figure S4(Supporting Information). C) Example of the decomposition of the spectra in the amide I′ window using the second derivative method.^[^ ^12^ ^]^ Black line, the experimental trace; magenta dashed line, the fitting curve. A series of spectra were acquired at various NaCl concentrations. D) Integrated intensities of the decomposed peaks at 5–100 mm NaCl concentrations. E,F) CH stretching window at the same 5–100 mm NaCl concentration range.

The results described in Section 2.1 inspired us to investigate the intrinsic property of SSB to determine whether SSB undergoes a structural transition in NaCl solutions to form two distinct conformations. We used Fourier transform infrared (FTIR) spectroscopy to examine the dynamics of SSB in NaCl solutions at various concentrations in the absence of ssDNA (**Figure** **2**A,B: Figure S4, Supporting Information). The wide peaks in the FTIR spectra were decomposed using the well‐known second derivative analysis method.^[^ ^12^ ^]^ (Figure 2C,E). We found that the backbone conformations (captured in the amide Iʹ region between 1600–1720 cm^−1^) remained largely consistent (Figure 2D), whereas the amino acid side chain conformations (within the CH stretch region from 2880–3000 cm^−1^) changed with increasing NaCl concentrations (Figure 2F). To confirm this result, we used two‐dimensional infrared (2D IR) spectroscopy to probe the dynamics within the CH stretch region (Figure S5, Supporting Information).^[^ ^13^ ^]^ Our results indicate that SSB underwent structural changes that were correlated with amino acid side chains rather than the protein backbone.^[^ ^14^ ^]^

### 2.3 Molecular Dynamics Simulations

**Figure 3:**
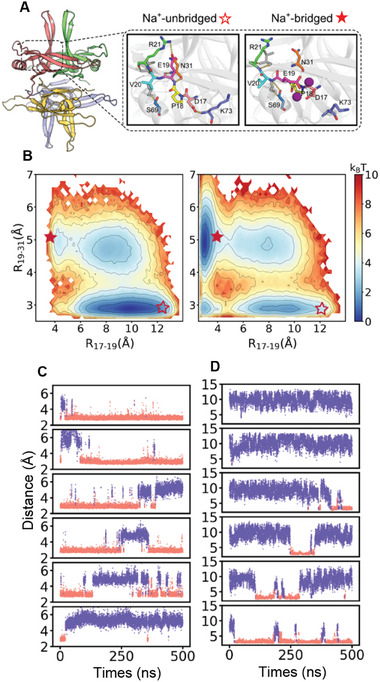
Molecular dynamics simulations. A) Structure of an SSB tetramer. The zoom‐ins show the hydrogen bond networks formed by the surface residues at low (0 m, left) and high (0.5 m, right) NaCl concentrations. B) 2D free energy profiles along the reaction coordinate R17–19 and R19–31 at low (0 m, left) and high (0.5 m, right) NaCl concentrations. Open and solid stars indicate the Na^+^‐unbridged and Na^+^‐bridged states, respectively. (C, D) Representative trajectories showing the distances R19–31 C) and R17–19 D) at different NaCl concentrations. Trajectories (top to bottom) correspond to simulations at NaCl concentrations of 0.00, 0.02, 0.05, 0.10, 0.20, and 0.50 m.

We carried out all‐atom molecular dynamics (MD) simulations of an SSB tetramer at different NaCl concentrations (**Figure** **3**). At low NaCl concentration (≤20 mm), the E19 residue of SSB tended to form a salt bridge with R21 and a hydrogen bond with N31 (Figure 3A, middle), and the sidechain of D17 formed a hydrogen bond with K73. At high NaCl concentration (≥200 mm), more than one sodium ion chelated with E19 and D17 (Figure S6, Supporting Information), leading to the reorientation of the two sidechains and rearrangement of the hydrogen bond network formed by the surrounding residues (Figure 3A, right). The hydrogen bond network rearrangement involved a free energy barrier, which therefore exhibited a cooperative two‐state feature as indicated by the free energy profiles (Figure 3B) and MD trajectories (Figure 3C,D). The electrostatic interaction arising from the Na^+^ binding was the major driving force of the reconfiguration of the hydrogen bond network, which occurred in all four subunits of SSB. The two main conformational states sampled by the simulations at the low and high salt concentrations are referred to as the “Na^+^‐unbridged” and “Na^+^‐bridged” states, respectively. The free energy of the Na^+^‐unbridged state increased at the higher NaCl concentrations (Figure 3B, open stars) in the conformational space characterized by the reaction coordinates R17–19 and R19–31 (which describe the shortest distances between two residues), whereas the free energy of the Na^+^‐bridged state decreased (Figure 3B, solid stars). At intermediate salt concentrations, switches occurred between the Na^+^‐bridged and Na^+^‐unbridged states and the relative stability of the two states shifted (Figure 3C,D; Figures S7 and S8, Supporting Information). The structural transition occurred mainly on the SSB surface (Figure S9, Supporting Information). We compared the sampled structures obtained by the all‐atom MD simulations with the corresponding crystal structure from the SSB–ssDNA complex.^[^ ^15^ ^]^ and found that the backbone conformation of the SSB core underwent only negligible changes in the MD simulations (Figure S10, Supporting Information).

**Figure 4:**
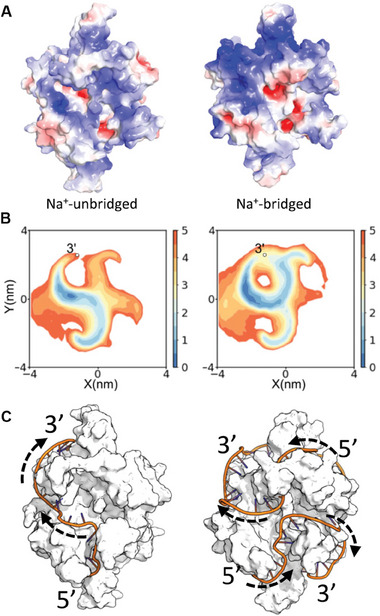
Electrostatic potential landscapes on the protein surface and their impacts on the wrapping of ssDNA on SSB. A) Electrostatic potential landscapes on the SSB surface sampled by the all‐atom molecular dynamics simulations at low (0 m, left) and high (0.5 m, right) NaCl concentrations. Blue, positive potential. B) 2D free energy profiles showing the locations of a 19‐nt ssDNA projected on the X–Y plane at low (0.01 m, left) and high (0.3 m, right) NaCl concentrations. The profiles were obtained using the 19‐nt ssDNA as a probe for the surfaces in (A). The potential of mean force is given as k_B_T. C) Distinct wrapping modes of a 70‐nt ssDNA on the surfaces in (A) at low (0.01 m, left) and high (0.3 m, right) NaCl concentrations. For clarity, the wrapping traces of ssDNA obtained from the coarse‐grained simulations are superimposed on the corresponding all‐atom structures of SSB.

The sodium chelation and the induced structural transition were accompanied by modification of the electrostatic potential landscape on SSB (**Figure** **4**A), as indicated by the overall enhancement of positive potential and the change of the distribution pattern. In the magnetic tweezers assay, this change was probed using an oligonucleotide via the ssDNA–SSB interaction. To produce the molecular events that occurred during the binding of an oligonucleotide to SSB, we constructed a coarse‐grained model and parameterized the electrostatic interactions based on the electrostatic potentials extracted from the all‐atom MD simulations at low and high NaCl concentrations (see Methods). This approach allowed us to effectively model the redistribution of the electrostatic potential on the SSB surface. We conducted coarse‐grained MD simulations of the interaction between a 19‐nt ssDNA and an SSB tetramer with the 3′‐end of the ssDNA being anchored at the SSB surface (see Figure S11, Supporting Information for details), which allowed converged sampling of the local wrapping pathways. We used a short spring to replace the first nucleotide in the simulation so that the total length was equivalent to 20 nt, which is similar to the length used in the magnetic tweezers assay. In the coarse‐grained MD simulations, the water effect was included implicitly using the Debye–Hückel continuum solvation model that is used widely in MD simulations of protein–DNA interactions at different salt concentrations.^[^ ^16^ ^]^ The calculated 2D free energy profiles (Figure 4B) indicate locations of the ssDNA projected on the X–Y plane of a local coordinate system illustrated schematically in Figure S12 (Supporting Information). The free energy profile had an S‐like shape at low NaCl concentrations (Figure 4B, left), and an O‐like shape at high NaCl concentrations (Figure 4B, right). This difference in the profile topology further confirms that the structural transition on the surface of SSB had significant effects on its interaction with ssDNA.

The crystal structure of the ssDNA–SSB complex suggests that the 65‐nt binding mode features bending of ssDNA around the central L_45_ loop, whereas such bending of ssDNA is absent in the 35‐nt binding mode.^[^ ^15^ ^]^ Indeed, the free energy profile at the high NaCl concentration (Figure 4B, right) shows that the ssDNA had a high probability of wrapping around the central L_45_ loop, which is a prerequisite for the 65‐nt binding mode observed experimentally at high salt concentrations.^[^ ^4b^ ^]^ We believe that the structural transition on the surface of SSB may contribute significantly to the transition of the ssDNA binding mode on SSB. To confirm this idea, we simulated the binding of a 70‐nt ssDNA to an SSB tetramer (Figure 4C; Figure S13, Supporting Information) and found that ssDNA tended to wrap along SSB more extensively and occupy more subunits (3 or 4 subunits) at the high NaCl concentration compared with the tendency at the low NaCl concentration (1 or 2 subunits).

## 3 Conclusion

Cationic and anionic residues on the surface of proteins often form hydrogen bond networks and play significant roles in shaping protein–ligand interactions. Our comprehensive approach showed that monovalent salts such as NaCl not only exhibit non‐specific electrostatic screening effects but also alter the organization of charged residues on the protein surface. Monovalent salts, even at moderate concentrations, can induce two‐state structural transitions on the protein surface. The magnetic tweezers analysis and MD simulations both indicated that two structural states can exist in equilibrium and in different proportions and that one was gradually phased out as the salt concentration increased. The two‐state structural transition contributed significantly to the transition of the ssDNA binding mode on SSB. Indeed, the simulations showed that 70‐nt ssDNA wrapped along the SSB more extensively and occupied more subunits on SSB at the high NaCl concentrations than it did at the low NaCl concentrations (Figure 4C). The cooperative rearrangement of the amino acid side chains, along with the associated alternation of electrostatic potential landscape on the protein surface, was instrumental in inducing the enigmatic transitions between the wrapping modes of ssDNA on SSB. Considering the ubiquity of protein–ligand interactions, many of which are electrostatically driven, we expect that surface structural transitions will be identified in more proteins in future research.

## 4 Experimental Section

### The DNA Constructs and the SSB Protein

The DNA constructs for the MT assay contain three parts: a 2300‐bp dsDNA and a 1000‐bp dsDNA as handles and a 20‐nt ssDNA (Poly dT) as the SSB binding site.^[^ ^17^ ^]^ The *E. coli*. SSB protein was purchased from Sigma‐Aldrich (S3917). SSB was stored in an SSB storage buffer (20 mm Tris‐HCl, pH 8.0, 0.5 m NaCl, 0.1 mm EDTA, 0.1 mm DTT, 50% glycerol). Before injected into the chamber, SSB was diluted by the reaction buffer (20 mm Tris‐HCl, pH 8.0, 20–200 mm NaCl).

### The Magnetic Tweezers Assay

A flow chamber was made of a coverslip and a slide. The coverslip was thoroughly cleaned and then silanized with Sigmacote (Sigma‐Aldrich). Its surface was modified by anti‐digoxigenin and blocked by a passivation buffer (10 mg ml^−1^ BSA, 1 mm EDTA, 10 mm pH 7.4 phosphate buffer, 10 mg ml^−1^ Pluronic F127 surfactant (Sigma‐Aldrich)). The chamber was mounted on an inverted microscope (Olympus IX71, 100× oil immersion objective, NA 1.45). The magnetic beads (2.8 µm diameter, streptavidin coated, Invitrogen) were treated by a passivation buffer overnight and washed by PBS (pH 7.4) before use. After checking the single DNA linkage by stretching DNA and observing the length change after twisting, the SSB solution was injected and incubated for 10 min. The length of DNA was continuously recorded under different forces.

### Calculation of the Binding Free Energy Using the MT Data

The binding free energy stands as the most employed metric for quantifying biomolecular interactions. It was often calculated by *Δ G* ^0^ =   − *k*_*B*_ *T*ln *K* where *k_B_* is the Boltzmann constant, *T* the temperature, and *K* the intrinsic equilibrium reaction constant for a ligand *L* binding to a substrate *S* to form a complex *LS*, $L+S\underset{k-}{\overset{k+}{⟷}}\mathrm{LS}$, where *k* ^+^ and *k* ^−^ are the binding and unbinding rate, respectively, from which one can calculate the reaction constant *K* = *k* ^+^/*k* ^−^.

Single‐molecule techniques were powerful tools to measure protein‐DNA interactions.^[^ ^18^ ^]^ In single‐molecule assays, the reaction kinetics can be regulated by external force, as these reactions frequently result in configurational changes.^[^ ^19^ ^]^ When force was applied slowly enough, allowing the system to maintain quasi‐static conditions, the bimolecular reaction constant, as observed, undergoes a transformation according to

(1) $$K_{obs}=K^{*}\;\exp\left(-\Delta \Delta G/k_{B}T\right)$$

where

(2) $$\Delta \Delta G\;=\int Fdx-\Delta G_{stretch}$$

and K* = [*L*]*k* ^+^/*k* ^−^ is the pseudo‐rate constant.^[^ ^20^ ^]^ The pseudo‐rate constant was proportional to ligand concentration [L] because, in single‐molecule assays, one usually considers the probability of finding a substrate S in a certain state rather than its concentration.^[^ ^20^ ^]^ The integration in Equation (2) represents the work done by the force and ΔG*_stretch_* takes into account the fact that the force not only changes the equilibrium but also increases the elastic energy of the system.^[^ ^19a,c^ ^]^ When a critical force Fc is exerted so that *K*_obs_/[*L*] = 1, the binding free energy ΔG_0_ at zero force is equal to −ΔΔG which can be readily calculated using Equation (2).

### Fourier Transform IR and Time‐Resolved 2D IR Spectroscopy

The FTIR spectra were collected in a Bruker Tensor II FTIR spectrometer with 64 scans at a resolution of 4 cm^−1^. The original SSB solution was mixed in a 1:3 volume ratio with the D_2_O buffer, which was the same as that used in the MT assay except that H_2_O was replaced with D_2_O. The sample was let stand for 24 h in a 10 °C environment for the exchange of H and D. It was then washed 3 times with the D_2_O buffer by ultrafiltration. The final concentration of SSB was 5 mg ml^−1^. The proteins in different salt solutions were held in a sandwiched structure with two CaF_2_ windows separated by a 100 µm path length Teflon spacer. The whole system was sealed and continuously flushed out with dry air to reduce the air humidity.

The same samples were then measured by using the 2D IR on a system described before.^[^ ^21^ ^]^ Briefly, 1 kHz pulse trains of 90 fs centered at 800 nm with 3.5 mJ output were generated by a Ti:sapphire regenerative amplifier (Spectra‐Physic, Spitfire) seeded with an oscillator (Spectra‐Physics, spitfire). ≈70% of the output was used to pump the commercially automated optical parametric amplifier TOPAS (Spectra‐Physic, Spitfire). The generated signal and idler were led through a difference frequency generation module (Spectra‐Physic, Spitfire), producing mid‐IR pulses centered at 5 µm with a 350 cm^−1^ spectral width and 35 µJ output energy. After the TOPAS, ≈5% of the mid‐IR radiation was split off by a 3° CaF_2_ wedge to become the probe beam, the remaining 95% of the mid‐IR pulse was transmitted through the wedge and introduced into a home‐built pulse‐shaper system based on a germanium acousto‐optic modulator (AOM, Isomet–LS600–1109–W).^[^ ^21^ ^]^ After the chirp compensation and spectra calibration, the modulated two pulses from the pulse shaper passed a translation stage producing a waiting‐time T and spatially overlapped with the probe beam at the sample position in pump‐probe beam geometry. The produced signals were collected by the 64‐channel detector (FPAS‐0144, Infrared Systems Development) combined with 300 mm focal length spectrometer (SP2300i, Princeton Instrument).

### All‐Atom Molecular Dynamics Simulations of Free SSB

To study the structural transition on the surface of SSB, an all‐atom MD simulation of SSB was performed under various salt concentrations. The coordinates of the atoms for the initial structure of SSB were extracted from the Protein Data Bank (PDB entry: 1EYG),^[^ ^15^ ^]^ with the DNA segments removed. The missing residues were reconstructed by using Modeler software.^[^ ^22^ ^]^ The protein was solvated by TIP3P ^[^ ^23^ ^]^ in water in a cubic box. Na^+^ and Cl^−^ were added into the simulation box to neutralize the simulation systems and to model the corresponding salt concentrations. The protonation states of the amino acids at neutral pH values were used. The titratable groups in the sidechains of the amino acids Tyr, Lys, and Arg were protonated. The titratable groups in the sidechains of the amino acids Asp and Glu were deprotonated. Additionally, the amino acid His was assigned a protonated state at the epsilon nitrogen atom. The MD simulations were performed by the software Gromacs2021.3.^[^ ^24^ ^]^ with the AMBER14SB.^[^ ^25^ ^]^ force field. Each system contains ≈102 000 atoms. The solvated systems were first minimized for 50 000 steps, which was followed by relaxation in the NVT ensemble at 300 K for 2.0 ns. The obtained systems were further relaxed for another 2.0 ns in the NPT ensemble at 300K and 1.0 atm. In the above relaxation simulations, positional restraints with harmonic potential were applied to all the heavy atoms of the protein, with the force constant of 1000 kJ mol^−1^ nm^−2^. Additional relaxation simulations were then performed for another 2.0 ns in the NVT and NPT ensemble without applying positional restraints. Finally, production simulations at 300 K and 1.0 atm were performed for 500 ns. 15 independent MD simulations were performed with different initial conditions, and the snapshots of the first 100 ns were omitted in the calculations of the average values and standard deviations. It was worth noting that the protonation states of the titratable residues may be dynamically modified due to the conformational changes and the electrostatic potential redistribution. Reasonably describing such charge regulation effect may need constant pH molecular dynamics, and it was beyond the capability of the simulation methods used in this work. It will be interesting to investigate the effects of charge regulation in the ssDNA–SSB interactions and wrapping in future studies using constant pH molecular dynamics simulations.

### Coarse‐Grained Model and Molecular Simulations of Binding between SSB and ssDNA

Because the timescale involved in the wrapping of ssDNA along the SSB surface was inaccessible for all‐atom MD simulations due to computational complexity, coarse‐grained molecular simulations were performed to study the binding between SSB and ssDNA. In the coarse‐grained model, each residue of SSB was represented by a single particle centered on its C_α_ atom. The interactions between the residues were described by the AICG^2+^.^[^ ^26^ ^]^ energy function, which was a structure‐based energy function developed based on the energy landscape theory and multiscale strategy.^[^ ^26^, ^27^ ^]^ The crystal structure (PDB entry: 1EYG).^[^ ^15^ ^]^ was used as the reference structure in constructing the structure‐based energy function. The ssDNA was described by the 3SPN.2 ^[^ ^28^ ^]^ model developed by de Pablo and coworkers. In the 3SPN.2 model, each nucleotide was represented by three beads, located at the centers of mass of the phosphate group, sugar group, and base group, respectively. The energy function of the 3SPN.2 include the terms of local interactions, base stacking interactions, base‐pairing interactions, excluded volume interactions, and electrostatic interactions. The 3SPN.2 model can well reproduce the persistence length and flexibility of dsDNA and ssDNA at different salt concentrations.^[^ ^28^ ^]^

The electrostatic interaction between the negatively charged phosphate groups of ssDNA and the charged residues of SSB plays a dominant role for the binding between the ssDNA and SSB. In addition, the base of ssDNA may also contribute to the stabilization of the ssDNA‐SSB complex, dominantly through hydrophobic interactions with the aromatic residues.^[^ ^21^ ^]^ Therefore, the interactions between ssDNA and SSB were described by the following energy function:

(3) $$E_{SSB-ssDNA}=E_{SSB-ssDNA}^{B}\;+E_{SSB-ssDNA}^{P}+E_{SSB-ssDNA}^{EXV}$$

where$E_{SSB-ssDNA}^{B}$ and $E_{SSB-ssDNA}^{P}$ represent the inter‐molecule interactions involving base and phosphate groups of ssDNA, respectively. $E_{SSB-ssDNA}^{EXV}$ represents the excluded volume effect of the inter‐molecule bead pairs. In this work, a structure‐based energy function was used to describe the interactions between ssDNA and SSB,^[^ ^29^ ^]^ and the term $E_{SSB-ssDNA}^{B}$ was given by

(4) $$E_{SSB-ssDNA}^{B}=\sum_{ij}\varepsilon \left[5{\left(\frac{r_{i}^{0}}{r_{ij}}\right)}^{12}-6{\left(\frac{r_{i}^{0}}{r_{ij}}\right)}^{10}\right]\;$$

where *r_ij_* is the distance of the residue *i* in SSB and the base *j* in ssDNA. $r_{i}^{0}$ is the distance between the residue *i* and its nearest base in the crystal structure. As all the nucleotides of SSB have the same identity (Poly T) in this work, they share the same $r_{i}^{0}$ value for a given residue *i*. Here, the summation index *i* runs over all the residues forming native contacts with bases in the crystal structure of SSB, and the summation index *j* runs over all the bases in ssDNA. The coefficient *ε* in the above energy function determines the strength of the inter‐molecule interactions and was set to 0.2325 kcal mol^−1^ in this work.

The electrostatic interactions between the negatively charged phosphate groups and the protein residues, that is, $E_{SSB-ssDNA}^{B}$, were described by the Debye‐Hückel model.^[^ ^30^ ^]^ The results of all‐atom MD simulations showed that specific Na^+^ binding tends to induce sidechain reorientation of the surface residues, which therefore leads to redistribution of electrostatic potential on the SSB surface. In order to reasonably describe the effect of redistribution of electrostatic potential on SSB surface due to Na^+^ binding induced sidechain reorientation, the partial charges of the coarse‐grained beads were optimized according to the electrostatic potentials calculated based on the structures of free SSB obtained from all‐atom MD simulations at high salt concentration (500 mm) and low salt concentration (0 mm) by using the RESPAC method.^[^ ^31^ ^]^ All the coarse‐grained MD simulations were performed by CafeMol 3.0 ^[^ ^30^ ^]^ by Langevin dynamics with a temperature of 300 K.

Coarse‐grained simulations were performed for the binding between SSB and 19 nt ssDNA at different salt concentrations with the above two protein models with different partial charges. In the simulations, the SSB protein and the 19 nt ssDNA were placed in a cubic box of 170 Å × 170 Å × 170 Å. In order to reduce the computational complexity, the 3′ terminus of the 19 nt ssDNA was anchored on the corresponding site at one of the OB domains of SSB by adding harmonic restraint potential on the two terminal base groups. The spring constant was set to 0.3 kcal mol^−1^ Å^−2^. Therefore, the location of 5′ terminus and other parts of the 19 nt ssDNA can be used to represent the wrapping mode. 30 independent simulations were performed with 3.5 × 10^8^ MD steps for each salt concentration for good convergence with the time step of 0.4τ, with τ being the reduced time unit used in CafeMol software. The snapshots of the first 5 × 10^7^ MD steps were omitted in the statistical analysis. The above CG model enables molecular simulations of the full binding events of ssDNA to SSB and captures implicitly the effect of the salt‐induced structural transitions of free SSB. However, due to the elimination of the degree of freedom of the sidechains, the explicit treatment of the sidechain rearrangement arising from the ssDNA binding was lacking, which can also contribute to the ssDNA–SSB interactions. In the calculation of the 2D free energy profiles in Figure 4B, the reaction coordinates (X, Y) defined by the local coordinate system are illustrated schematically were used in Figure S12 (Supporting Information).

## Conflict of Interest

The authors declare no conflict of interest.

## Supporting information

Supporting Information

## Data Availability

The simulation trajectories and related parameter files are uploaded to GitHub (https://github.com/wuyichao71/SSB_simulation).
