# Supplementary material for: Sodium Ion‐Induced Structural Transition on the Surface of a DNA‐Interacting Protein
Source: Adv Sci (Weinh). 2024 Sep 20;11(42):2401838. doi: 10.1002/advs.202401838 (PMC11558118; doi:10.1002/advs.202401838)
Supplement: Supplementary file 1 — Supporting Information [file ADVS-11-2401838-s001.docx]

Supporting Information

Sodium Ion-Induced Structural Transition on the Surface of a DNA-Interacting Protein

*Chunhua Xu^+^, Yue Lu^+^, Yichao Wu^+^, Shuaikang Yuan^+^, Jianbing Ma, Hang Fu, Hao Wang, Libang Wang, Hao Zhang, Xuan Yu, Wei Tao, Chang Liu, Shuxin Hu, Yi Peng, Wenfei Li*, Yunliang Li*, Ying Lu*, and Ming Li**


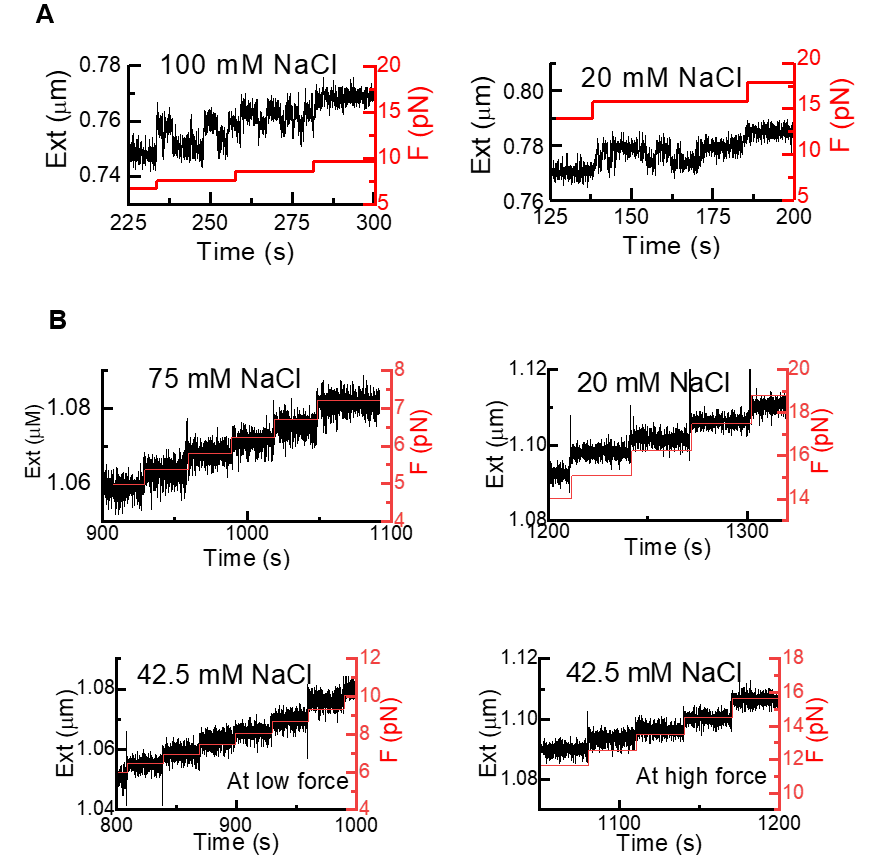


Figure S1. Two control experiments. (A) The binding/unbinding observed by MT using a 70 nt ssDNA. Typical time traces of the DNA extension (black lines) at different forces (red lines) at 100 mM (left panel) and 20 mM NaCl (right panel), respectively. (B) A control experiment was performed without SSB using the DNA construct (a 20-nt ssDNA with two dsDNA handles). In the absence of SSBs, the DNA substrate does not exhibit the jump phenomenon under a variety of conditions, including high or low salt concentrations, or intermediate concentrations accompanied by low or high forces.


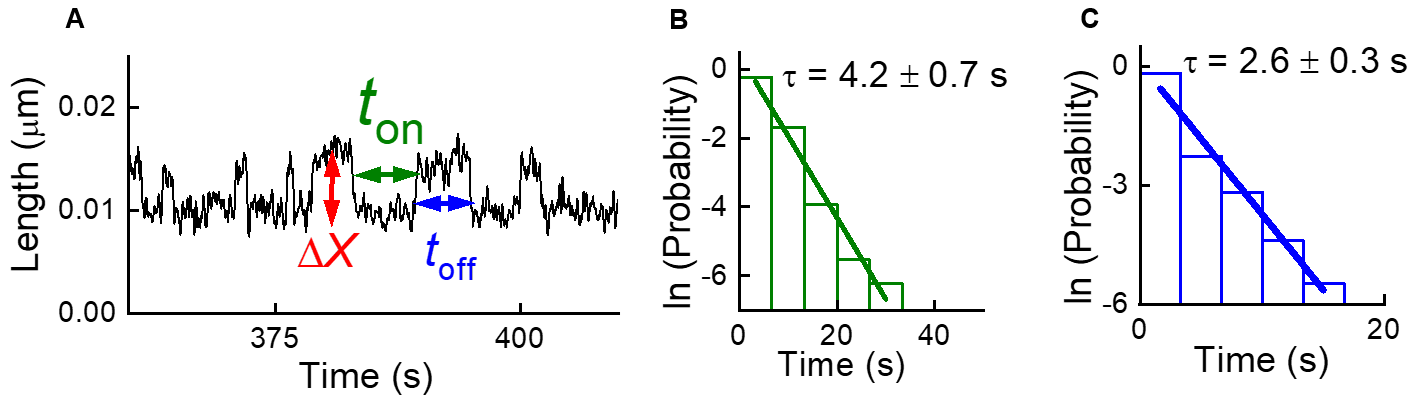


Figure S2. The binding/unbinding dwell times. (A) An example of the binding/unbinding time traces at [NaCl] = 75 mM and [SSB] = 10 nM. The large values represent the unbound state and the small values the bound state. (B) Dwell time distribution of the bound state. (C) Dwell time distribution of the unbound state.


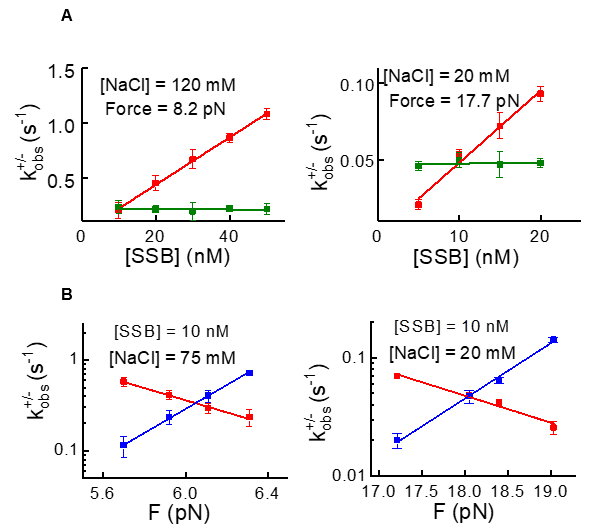


Figure S3. The binding/unbinding rates calculated according to the dwell times. (A) Binding (red)/unbinding (green) rates vs [SSB] at [NaCl] = 120 mM at Force = 8.2 pN (left panel) and [NaCl] = 20 mM at Force = 17.7 pN (right panel). The binding rate is proportional to [SSB]. The unbinding rate is independent of [SSB]. (B) Binding (red)/unbinding (blue) rates vs force at [SSB] = 10 nM with [NaCl] = 120 mM in the left panel and [NaCl] = 20 mM in the right. The critical force *F*_c_ was determined from the crossover of the two force-dependent curves. [SSB] = 10 nM.


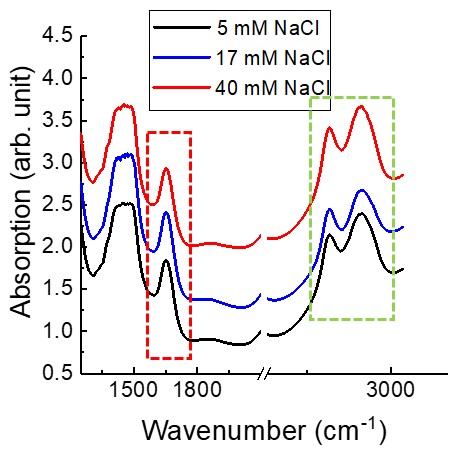


Figure S4. FTIR spectra of SSB at [NaCl] = 5, 17 and 40 mM in D_2_O. The red rectangle marks the amide Iʹ window between 1600–1720 cm⁻¹. The green rectangle marks the CH stretch window between 2880–3000 cm⁻¹.

**
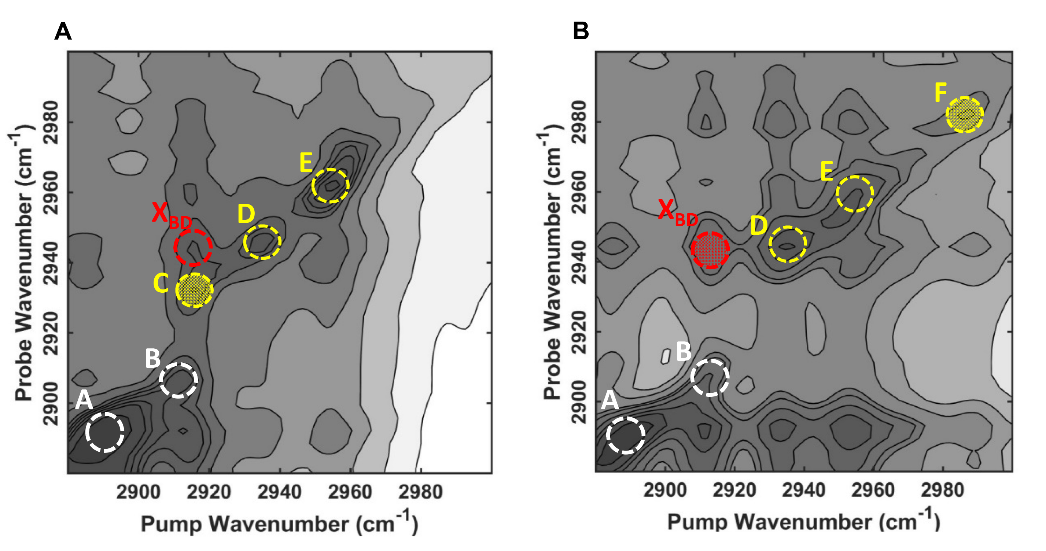
**

**Figure S5.** 2D IR spectra in the CH stretch window of SSB in two NaCl solutions. (A) [NaCl] = 5 mM. (B) [NaCl] = 40 mM. The time delay between the pump and probe is 0 fs. The diagonal peaks A and B (white circles) correspond to the symmetric CH stretch modes; the diagonal peaks C, D, E and F (yellow circles) correspond to the asymmetric CH stretch modes. The cross peaks arise from spatial correlations between the different conformational elements of the sidechains. The transparent full circles mark the peaks that undergo significant changes when the salt concentration is changed. A striking observation is the change of the diagonal peak C from clear visibility in the 5 mM NaCl solution to being overshadowed by the nearby cross peak X_BD_ in the 40 mM NaCl solution. The intensity of the cross peak X_BD_ is markedly elevated in this solution. In accordance with this, the peak D's intensity augments in the higher salt concentration, while the peak F, invisible at 5 mM NaCl, becomes clear at 40 mM NaCl.


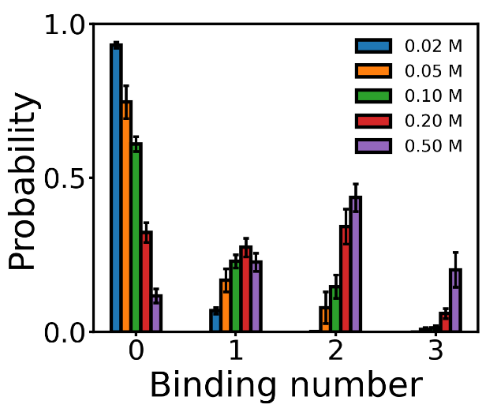


Figure S6. Probabilities of the number of sodium ions chelated by D17 and E19 at different salt concentrations from the all-atom MD simulations. One can see that the two-sodium chelated structure is dominant at high salt concentrations. The error bars represent the standard deviation calculated based on the structures of all the four subunits of SSB from 15 independent MD simulations. A sodium ion is considered to be chelated when the closest distances to the heavy atoms of the D17 and E19 are less than 3.0 Å. In calculating the probability, the snapshots of the first 100 ns were omitted.


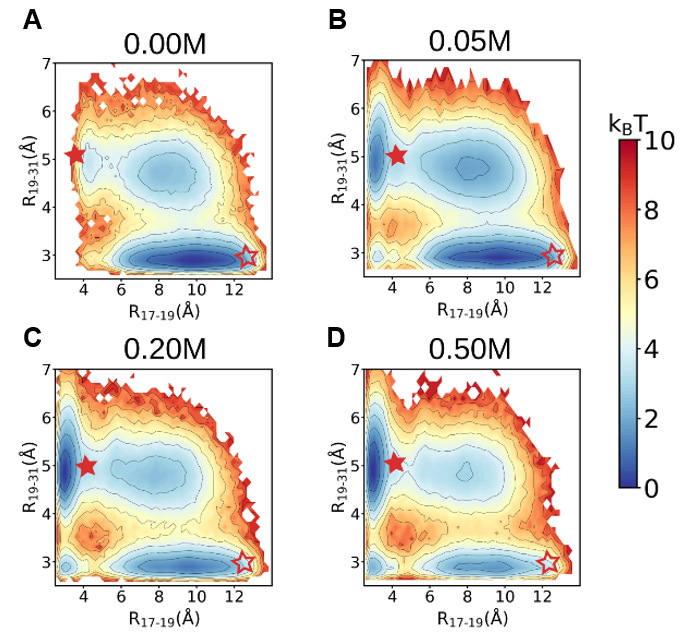


Figure S7. Two-dimensional free energy profiles along the reaction coordinates R_17-19_ and R_19-31_ at different salt concentrations. The reaction coordinate R_17-19_ (R_19-31_) represents the closest distance between the heavy atoms of the D17 and E19 (E19 and N31). The open and solid stars label the “Na^+^-unbridged” and “Na^+^-bridged” states, respectively.


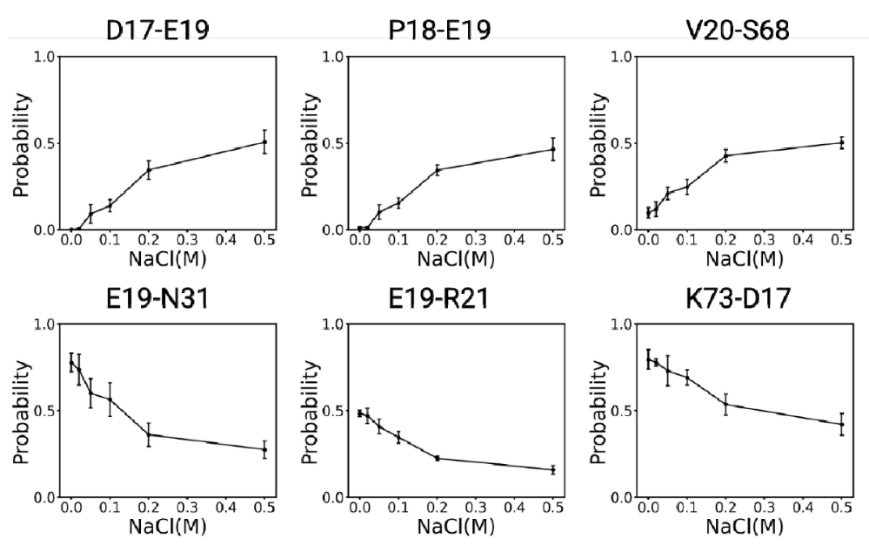


Figure S8. Probabilities of contact formation between the residue pairs as a function of salt concentration. The displayed are the probabilities of contact formation for the corresponding residue pairs at different salt concentrations. The error bars of the probabilities represent the standard deviation calculated based on the structures of all the four subunits of SSB from 15 independent MD simulations, with the snapshots of the first 100 ns being omitted. A contact is formed when the closest distance between the heavy atoms of the two residues is less than 3.5 Å.


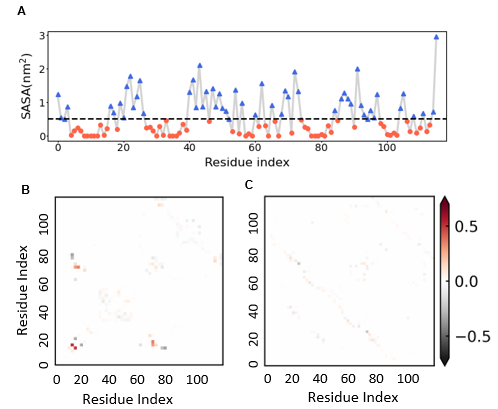


Figure S9. Salt-induced structural transition mainly occurs on the SSB surface. (A) Solvent accessible surface area (SASA) for each of the residues of SSB monomer. The dash line represents a threshold value (0.5 nm^2^) dividing the buried residues and surface residues. (B, C) Differences of contacting probabilities of the residue pairs between the low-salt case and high-salt case for the surface residues (B) and buried residues (C), respectively. Compared to the surface residues, the conformational change of the buried residues is much minor with the increasing of salt concentrations.


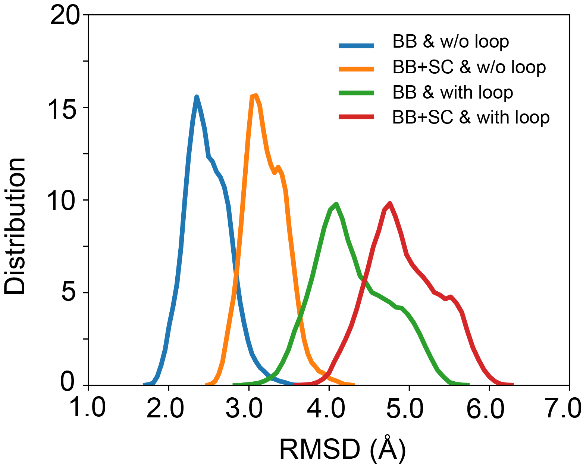


**Figure. S10.** Distribution of the root mean square deviation (RMSD) of the SSB tetramer structures sampled from the all-atom MD simulations with respect to the reference structure extracted from the crystal structure of ssDNA-SSB complex. The RMSD values were calculated for the backbone atoms of the SSB core without including the flexible loops (blue, BB & w/o loop), the backbone and the sidechain atoms of the SSB core without including the flexible loops (orange, BB+SC & w/o loop), the backbone atoms of the whole SSB (green, BB & with loop), and the backbone and the sidechain atoms of the whole SSB (red, BB+SC & with loop). All the sampled trajectories of the all-atom MD simulations at the salt concentration of 0.02 M were used in the calculation of RMSD distribution. One can see that the conformational change of the backbone atoms of the SSB core region is minor in the all-atom MD simulations compared to the crystal structure. The sidechain and the flexible loop regions have signigicant conformational fluctuations in the all-atom MD simulations.


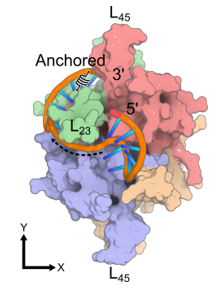


Figure S11. The simulation setup for the system with the 3' end of a 19 nt ssDNA anchored at the surface of the SSB as indicated in the figure. We used a short spring to replace the first nucleotide in the simulation so that the total length is equivalent to 20 nt like that used in the MT assay.


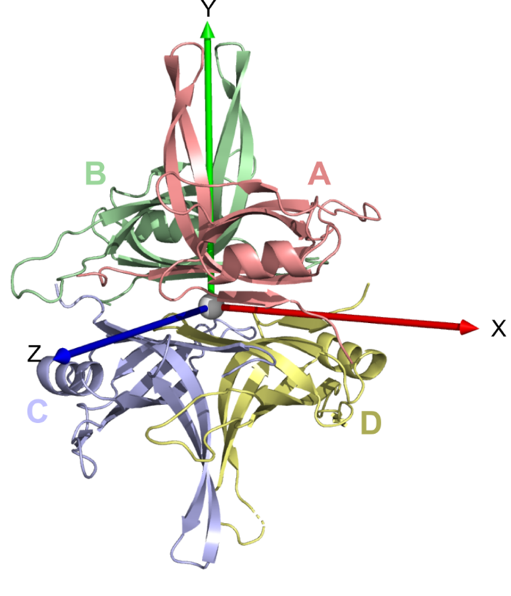


Figure. S12. Definition of the local coordinate system of SSB. The center of mass of the whole SSB is defined as the origin. The Y axis points from the center of mass of the chains C and D to the center of mass of the chains A and B. Then we defined a vector pointing from origin to the center of the residue 34 in chain A and residue 34 in chain D, and the Z axis is defined by the cross product of this vector and the Y axis. The X axis is defined by the cross product of the Z axis and the Y axis. The coordinates of the coarse-grained beads of ssDNA along this local coordinate system are used to calculate the 2D free energy profile Figure 4B.


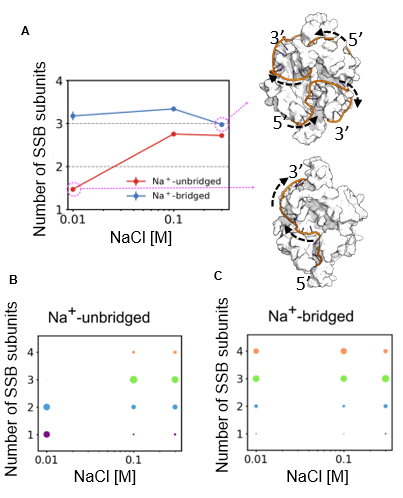


Figure S13. The CG MD simulations of the distinct wrapping modes of a 70 nt ssDNA on SSB. (A) Average number of the SSB subunits occupied by the 70 nt ssDNA (binding-site size) at different salt concentrations based on coarse-grained MD simulations for the Na^+^-unbridged state (red) and Na^+^-bridged state (blue). The error bars represent the standard deviation calculated based on 30 independent MD simulations. (B, C) Populations of the one-subunit occupied (~17nt), two-subunit occupied (~35nt), three-subunit occupied (~56nt), and four-subunit occupied (~65nt) ssDNA-SSB complexes for the Na^+^-unbridged state (B) and Na^+^-bridged state (C) at different salt concentrations. Probabilities of different complex structures are represented by the size of the colored circles. The simulations showed that the ssDNA tends to wrap along the SSB surface more extensively and occupy more sites (3 or 4 sites) on the SSB subunits at the high salt concentration compared to that at the low salt concentration (1 or 2 sites). The large difference between the results based on the Na^+^-unbridged state and Na^+^-bridged state suggests that the sodium ion induced structural transition has significant contribution to the distinct wrapping modes of ssDNA, in addition to the electrostatic screen effect. It is worth emphasizing that although SSB dominantly adopts Na^+^-unbridged and Na^+^-bridged structures at low and high salt concentrations, respectively, both structures may have significant populations at moderate salt concentrations. Therefore, at moderate salt concentrations, the results based on the two sets of partial charges represent two limit cases.

Table S1 The numbers of the Cl^－^ and Na^+^ ions added at different salt concentrations in the all-atom MD simulations.

| [NaCl] | 0.00 M | 0.02 M | 0.05 M | 0.10 M | 0.20 M | 0.50 M |
| --- | --- | --- | --- | --- | --- | --- |
|  |  |  |  |  |  |  |
| Cl^-^ | 8 | 21 | 39 | 71 | 133 | 322 |
| Na^+^ | 0 | 13 | 31 | 63 | 125 | 314 |
